# The cytochemical detection of oestrogen receptors in fine needle aspirates of breast cancer; correlation with biochemical assay and prediction of response to endocrine therapy.

**DOI:** 10.1038/bjc.1988.166

**Published:** 1988-07

**Authors:** R. A. Hawkins, K. Sangster, A. Tesdale, P. A. Levack, E. D. Anderson, U. Chetty, A. P. Forrest

**Affiliations:** University Department of Surgery, Royal Infirmary, Edinburgh, UK.

## Abstract

A total of 98 breast aspirates from patients with breast cancer have been fixed and stained for oestrogen receptors using the Abbott ERICA kit. In a preliminary series of 41 aspirates, cytochemical staining index (% cells staining x mean intensity) related to the receptor concentration determined biochemically on a subsequent biopsy with a correlation coefficient of +0.65. In a second series of 56 aspirates examined after lysis and cytocentrifugation, the correlation coefficient was +0.73. For 14 patients, the response of the primary tumour to endocrine therapy was assessed objectively by serial clinical and mammographic measurements (Forrest et al., 1986) and was found to relate strongly to the cytochemical staining of the initial aspirate. The potential and limitations of this technique are discussed.


*Br. J. Cancer* (1988), **58**, 77–80 © The Macmillan Press Ltd., 1988

### The cytochemical detection of oestrogen receptors in fine needle aspirates of breast cancer; correlation with biochemical assay and prediction of response to endocrine therapy

R.A. Hawkins, K. Sangster, A. Tesdale, P.A. Levack, E.D.C. Anderson, U. Chetty & A.P.M. Forrest

*University Department of Surgery, Royal Infirmary, Edinburgh EH3 9YW, UK.*

**Summary** A total of 98 breast aspirates from patients with breast cancer have been fixed and stained for oestrogen receptors using the Abbott ERICA kit. In a preliminary series of 41 aspirates, cytochemical staining index (% cells staining x mean intensity) related to the receptor concentration determined biochemically on a subsequent biopsy with a correlation coefficient of +0.65. In a second series of 56 aspirates examined after lysis and cytocentrifugation, the correlation coefficient was +0.73. For 14 patients, the response of the primary tumour to endocrine therapy was assessed objectively by serial clinical and mammographic measurements (Forrest *et al.*, 1986) and was found to relate strongly to the cytochemical staining of the initial aspirate. The potential and limitations of this technique are discussed.

The presence of oestrogen receptors within a breast tumour is now established as an index of both improved prognosis and increased likelihood of response to endocrine therapy (Hawkins, 1985). The generation of monoclonal antibodies against the human oestrogen receptor (Greene *et al.*, 1980) has permitted the development of commercial kits for both biochemical (ER-EIA) and histochemical (ER-ICA) assays by Abbott Laboratories. The results of the latter technique have been shown to correlate well with those of established biochemical, steroid-binding (DCC) methods on biopsies of solid tumour (King *et al.*, 1985; McLelland & Coombes, 1985; McCarty *et al.*, 1985; Hawkins *et al.*, 1986) and the technique has recently been extended to the assessment of receptor status in breast cancer cells collected by needle aspiration (McLelland & Coombes, 1985; Flowers *et al.*, 1985; McLelland *et al.*, 1987; Cavailles *et al.*, 1987; Weintraub *et al.*, 1987). We set out to examine the feasibility of using such a method and to compare the results of the technique with (a) those of our standard DCC assay on solid biopsy, established 15 years ago and (b) response to endocrine therapy in patients with large primary tumours.

### Methods and materials

### *Patients*

Aspirates were taken from patients presenting to the departmental clinic for the first time. The patients' ages ranged from 33 to 76 years. Aspirates were obtained by making approximately 10 passes through the tumour using a 21 gauge needle, mainly by two of us (PAL, EDCA). After preparation of smears for diagnostic cytopathology, residual material was used for fixation and cytochemical staining. In one subset of 14 patients with large tumours (>4 cm, $T_2/T_3$ and one $T_4$), the patient proceeded after aspiration to wedge biopsy for further biochemical and histological investigations prior to initiating endocrine therapy. The response of the primary tumour was monitored objectively by sequential clinical and mammographic measurements (Forrest *et al.*, 1986). Of these patients, five received the LHRH agonist Zoladex (ICI 118630), five aminoglutethimide, two 4-hydroxyandrostenedione and two tamoxifen. One patient was premenopausal, 12 were postmenopausal and one was male. Response was classed as regression, progression or stasis depending on a statistical evaluation of the changes in tumour size during a 12-week period (see Forrest *et al.*, 1986). The biochemical value of oestrogen receptor concentration was recorded in the patient's notes at the start of treatment but 'response' was decided by statistical evaluation, and in the absence of the knowledge of the cytochemical assay result.

### *Cytochemical assay*

In an initial series of 41 patients, aspirates were taken and fixed by a variety of techniques (A, B, C, D): in brief, these involved:

A. Preparation of a smear and fixation immediately in the clinic;
B. Preparation of a smear, storage on dry ice and fixation at the end of the clinic (essentially the method of Coombes *et al.*, 1987);
C. Collection of the aspirate in 'storage buffer' (PBS containing 50% glycerol v/v and 3 mM magnesium chloride), on ice, with cytocentrifugation and fixation at the end of the clinic;
and
D. Collection of the aspirate in saline/tissue culture medium (RPMI), on ice, with cytocentrifugation and fixation at the end of the clinic.

Ultimately a fifth method (E) was preferred and this was used to process a second series of aspirates from a further 56 patients. In this method (which is essentially that employed by Dr R.E. Leake, Department of Biochemistry, University of Glasgow), the residue of aspirate remaining after the preparation of the diagnostic smears was flushed gently from the syringe using tissue culture medium (0.2–1.0 ml RPMI). After addition of water (3:2 v/v) and brief (30 sec), gentle agitation to lyse red cells, further RPMI (12:5 v/v) was added and the cells were harvested by centrifugation for 15 min at 230 *g* in an MSE Mistral centrifuge. After removal of the supernatant fluid, the cells were resuspended in a suitable volume of medium (≧300 μl) and three 100 μl aliquots were cytospun for 10 min at 1,000 rpm (89 *g*) onto slides precoated with polylysine (Abbott) in a Cytospin 2 centrifuge. In order to ensure adhesion of the cells to the slides, these were left to air-dry for 2 min, prior to fixation and staining with the Abbott ERICA kit as we have described previously (Hawkins *et al.*, 1986). A single quality control slide (Abbott) was stained in each batch of aspirates.

Each slide was assessed independently by two observers (RAH, KS) for (a) the % cells staining, and (b) an average intensity of staining on a scale of 0, 1, 2 or 3 for all the cells in the cytospun area. After correction for any staining in the 'control' section (exposed to non-specific rat serum), a cytochemical staining index (average intensity of staining

Correspondence: R.A. Hawkins.
Received 21 January 1988; and in revised form, 13 April 1988.

x % cells staining/100) was calculated for the 'test' section (exposed to the anti-receptor antibody). Where there was significant disagreement between the observers (11.8% cases), the slides were accepted after rescoring by mutual consent (7.5%) or rejected as 'unassessable' (4.3%). Specimens showing very few cells, very large amounts of non-specific (control) staining or for which the formal histopathological report was 'benign' or 'inadequate' were also rejected. Specimens from patients on tamoxifen at aspiration were excluded.

In the second series of aspirates, for example, the 56 assessable specimens were derived from a total of 91 patients, exclusions being 3 because of tamoxifen treatment, 15 unassessable and 17 because the formal diagnostic smear was classified as 'acellular', 'benign' or 'lymphoma'.

*Biochemical assay of oestrogen receptor activity*

Solid tumour was obtained by biopsy or mastectomy, within 1–2 weeks of aspiration. This was used for the determination of oestrogen receptor activity by saturation analysis, with separation of free and bound hormone by dextran-coated charcoal (DCC) adsorption as described previously (Hawkins *et al.*, 1981). Specimens containing $\geq 5$ fmol oestrogen receptor sites $mg^{-1}$ soluble protein are considered 'receptor-positive'.

### Results

*1. Correlation between cytochemical and biochemical estimates of receptor activity*

In a preliminary series of 41 patients (of average age 55 years), 78% of the tumours contained receptors (range 5–789 fmol $mg^{-1}$ protein) by DCC assay and 73% showed cytochemical staining after fixation by a variety of methods (A, B, C, D). The degree of cytochemical staining ('staining index') in the tumour aspirate ranged from 0 to 2.9 and correlated moderately well ($r = +0.65$) with the receptor site concentration determined biochemically by DCC assay on the subsequent surgical biopsy specimen (not shown). In this series, however, cell losses during fixation and processing were a problem. A second series was therefore examined by method 'E'.

In the second series of patients (of average age 61 years), 48/56 (86%) of the tumours contained receptors (5–933 fmol $mg^{-1}$ protein) by DCC assay and 46/56 (82%) showed staining. The degree of staining in these cells, ranging from 0 to 2.9, showed a slightly stronger correlation with receptor site concentration by DCC assay ($r = +0.73$); the data are plotted in Figure 1. Similar correlations were observed when the relationship between % cells staining ($r = +0.69$) or staining intensity ($r = +0.78$) and receptor concentration were examined separately (not shown). It is noted, however, that under the conditions of assay employed, the tissues of receptor concentration less than 30 fmol $mg^{-1}$ protein showed relatively low levels of staining. The relationship between the qualitative categorisations of each assay result is summarised in Table I.

*2. Relationship of cytochemical staining for receptor and response to endocrine therapy*

Response of the primary tumour to endocrine therapy was assessed in 14 patients with large, but operable primary tumours. Six of the patients responded, 5 showed no clear change on therapy and 3 tumour progression (Table II).

With one exception, the tumours showing regression had a staining index of $\geq 1.40$; tumours showing stasis had indices between 0.12 and 1.05, and those tumours which progressed an index of 0.

Again in this small subseries, cytochemical staining correlated strongly with biochemical receptor site concentration ($r = +0.93$). Accordingly, biochemical receptor site concentration and cytochemical staining index both appeared to relate equally well to clinical response.

The same data are shown expressed in qualitative terms in the contingency Table III, where it can be seen that there is a significant relationship between receptor status and response for the cytochemical assay, though the relationship for the biochemical assay does not quite attain significance.

**Figure 1** Relationship between cytochemical staining (ERICA) of an aspirate and oestrogen receptor concentration determined by DCC assay in a subsequent biopsy for 56 patients with breast cancer. Staining index = average staining intensity x % cells staining/100 and is the mean of observations by two observers. The correlation coefficient (Spearman Rank Test) was +0.73.

**Table I** The qualitative relationship between cytochemical staining (ERICA) on an aspirate and oestrogen receptor activity determined biochemically by DCC assay in 56 patients with breast cancer

| *Biochemical receptor activity*[a] *(fmol mg⁻¹protein)* | *Cytochemical staining*[b] (−) | (+) |
|---|---|---|
| − | 7 | 1 |
| + | 3 | 45 |
| $X^2$ with Yates' correction = 25.57 | | |
| $P < 0.0005$ | | |

[a]Activity was taken as − when receptor site concentration was <5 fmol $mg^{-1}$ protein and + when $\geq 5$; [b]Staining was taken as − when the staining index = 0 and all values >0 were designated '+'.

**Table II** Relationship between cytochemical staining (ERICA) in breast tumur aspirates and response to endocrine therapy

| *Patient* | *Cytochemical staining index*[b] *(aspirates)* | *Biochemical fmol receptor sites mg*[1] *protein (biopsy)* | *Clinical response* |
|---|---|---|---|
| 1 | 2.90 | 174 | Regr[a] |
| 2 | 2.25 | 364 | Regr |
| 3 | 2.25 | 344 | Regr |
| 4 | 1.83 | 412 | Regr |
| 5 | 1.40 | 147 | Regr |
| 6 | 1.05 | 167 | Stasis |
| 7[c] | 0.55 | 91 | Stasis |
| 8 | 0.51 | 73 | Stasis |
| 9 | 0.49 | n.a. | Stasis |
| 10 | 0.12 | 77 | Stasis |
| 11 | 0.03 | 17 | Regr |
| 12 | 0 | 6 | Progression |
| 13 | 0 | 0 | Progression |
| 14 (male) | 0 | 0 | Progression |

[a]Regr = regression; [b]Staining index = staining intensity x % cells staining/100; [c]Early $T_4$ tumour, all other patients had $T_2/T_3$ tumours; n.a. = no biopsy available.

**Table III** Relationship between receptor status and response to endocrine therapy in 14 patients with large, primary tumours of the breast

| *Receptor status* | *Regression* | *Stasis* | *Progression* |
|---|---|---|---|
| Cytochemical staining[a] | | | |
| + | 6 | 5 | 0 |
| – | 0 | 0 | 3 |
| | | $P<0.0055$ | |
| Biochemical receptor activity[b] | | | |
| + | 6 | 5 | 1 |
| – | 0 | 0 | 2 |
| | | $P<0.066$ | |

[a]Staining was taken as – when the staining index = 0 and all values >0 were designated '+'; [b]Activity was taken as – when receptor site concentration was <5 fmol mg$^{-1}$ protein and + when ≥5; *P* values were derived from an exact test for trend.

### Discssion

The studies which we report here confirm the reports of others who have suggested that cytochemical staining of breast aspirates by the ERICA kit (1) accurately reflects the oestrogen receptor content of the tumour and therefore (2) accurately relates to the patient's clinical response to endocrine therapy. Despite the presence of a few tumours of moderate/higher receptor value showing a low staining index (<0.5, see Figure 1), there was a good correlation between cytochemical and biochemical assays. The reasons for the low degree of staining in a few tumours of higher biochemically determined receptor concentration are not fully resolved. Inspection of such specimens shows that the lower values of staining index derive almost entirely from the small percentage of the cells showing staining and not from a low intensity of staining. However, these specimens were not associated with either poorer cellularity or higher blood content than those showing a stronger correlation between cytochemistry and biochemistry. Nevertheless our finding of a correlation coefficient of +0.73 ($n=56$) for the quantitative relationship between staining on aspirates and receptor site concentration in a biopsy agrees well with the equivalent correlations of +0.74 ($n=33$) and +0.76 ($n=35$) reported by Flowers *et al.* (1985) and Cavailles *et al.* (1987) respectively. These correlations are higher than that of +0.48 ($n=95$) found by McLelland *et al.* (1987), but their series included some comparisons between ERICA staining and DCC assays on different tumour deposits. Weintraub and his colleagues (1987) compared ERICA staining in aspirates and frozen sections from the same tumour specimen and also found a correlation coefficient of +0.72 ($n=31$); however, there was a lesser correlation (+0.55) between aspirate staining and the concentration of biochemically determined receptor sites. In general, these correlations are of the same order of magnitude as those found by ourselves ($r=+0.87$, $n=34$, Hawkins *et al.*, 1986) and others, e.g. Andersen *et al.* (1986) ($r=+0.91$, $n=35$), McCarty *et al.* (1985) ($r=+0.79$, $n=62$) and King *et al.* (1985) ($r=+0.76$, $n=38$) when comparing ERICA staining in frozen or paraffin sections with the concentration of biochemically determined receptor sites in the same tumour specimen. In view of the heterogeneity of breast tumours with respect to oestrogen receptor concentration (e.g. Braunsberg *et al.*, 1975; Hawkins *et al.*, 1977; Senbanjo *et al.*, 1986), it may not be possible to attain a better correlation between assays on aspirates and those on excision biopsies.

The relationship between ERICA staining and response to endocrine therapy has been determined on only a small series of patients. Nevertheless, in our experience, the primary tumour model used gives a much more precise index of tumour response than do studies of metastatic disease: it allows receptor activity and response of the same tumour mass to be related. The relationship between staining and response is striking and could not occur by chance. These findings support the contention of Coombes *et al.* (1987) who, though using a slightly different technique for processing of the aspirates, demonstrated that cytochemical staining accurately predicts response to endocrine therapy in 60 patients with metastatic or locally advanced breast cancer. The present data are insufficient to allow selection of a clinically useful 'cut-off point' for the staining index, though McLelland & Coombes (1985) have previously suggested a value of 0.5 on a comparable scale.

It is to be noted that for one patient (Number 11), response was associated with a very low staining intensity: this woman was postmenopausal, treated by administration of LHRH agonist and showed a statistically significant regression. Pretreatment receptor levels were low by either cytochemical or biochemical techniques, though at subsequent mastectomy, a second biochemical assay showed a much higher receptor level (102 fmol mg$^{-1}$ protein).

Despite the potential value of the staining of breast aspirates by ERICA, it is important to consider five limitations to this method. Firstly, the method we have preferred does produce some morphological damage to the aspirated cells and would not permit interpretation of cell-type in all cases, though a concomitant smear, fixed directly, can easily be processed for each aspirate. Reduction of the centrifugation times and *g* forces to 5 min at 115 *g* for harvesting the cells and 4 min at 89 *g* for cytospinning has been found subsequently to lessen the damage. This technique differs from the more direct method of preparing a smear used by Coombes and his group. In our hands, however, we found that staining smears used much larger amounts of antibody, yielded much wider areas to assess microscopically, and gave problems, with the interpretation particularly of bloody smears, since red blood cells contain high levels of endogenous peroxidase. Secondly, in its present form, the assay may not be sufficiently sensitive to detect staining in some premenopausal women who, with low levels of receptor activity by biochemical assay (20–30 fmol mg$^{-1}$ protein) may nevertheless respond to endocrine therapy (Hawkins – unpublished, Nicholson, R.I. – personal communication). Thirdly, a minimum number of tumour cells (perhaps 20) is required as an adequate sample for assessment: although staining may be visible in a lesser number of cells, the small number of cells may be insufficient to take account of possible heterogeneity (R+ and R– cells). We would not agree with Flowers *et al.* (1985), who considered 5 cells as adequate. Fourthly, it is important that some measure of quality control be applied to the ERICA assays to ensure consistent sensitivity. Although we routinely processed and scored the control slide provided by Abbott, this single control may be inappropriately high for assessing samples with receptor levels as low as 20 fmol mg$^{-1}$ protein and we did detect some significant variations in staining of the control slide between assays. This may be of importance since our experience with advanced disease would indicate that 20 fmol mg$^{-1}$ protein is a suitable 'cut-off' for selecting patients for endocrine therapy. Lastly, since neither of us who scored the slides were pathologists, we confined our assessments to only those patients in whom carcinoma had been diagnosed by the breast cytologist on concomitantly prepared smears of the aspirated cells, and it may well be that a superior predictive index can be derived when the stained material is scored by an experienced breast cytopathologist.

We wish to thank Dr Robin Leake, University of Glasgow, for supplying details of his method for fixing breast aspirates and for useful discussions. Dr R.C. Coombes, St George's Hospital Medical School, London, also kindly made available details of his technique at an early stage. We are grateful to Drs T.J. Anderson, J. Lamb and E. McGoogan of the Department of Pathology for the cytopathological diagnosis on the aspirates, for selecting the appropriate area of solid tumour for DCC assay, and for advice and discussion of the problems involved in the cytochemical technique. Miss G. White, Scottish Cancer Trials Office, kindly advised on the statistical assessments.

This work was supported by a grant from the Scottish Home and Health Department to Drs R.A. Hawkins and R.E. Leake. Dr Levack and Miss Anderson received support from a Cancer Research Campaign grant to Prof. Sir Patrick Forrest.

### References

ANDERSEN, J., ORNTOFT, T. & SKOVGAARD POULSEN, H. (1986). Semiquantitative oestrogen receptor assay in formalin-fixed paraffin sections of human breast cancer tissue using monoclonal antibodies. *Br. J. Cancer*, **53,** 691.

BRAUNSBERG, H. (1975). Factors influencing the estimation of oestrogen receptors in human malignant breast tumours. *Eur. J. Cancer*, **11,** 499.

CAVAILLES, V., GARCIA, M., SALAZAR, G. & 4 others (1987). Immunodetection of oestrogen receptor and 52,000-Dalton protein in fine needle aspirates of breast cancer tumours. *J. Natl Cancer Inst.*, **79,** 245.

COOMBES, R.C., POWLES, T.J., BERGER, U. & 5 others (1987). Prediction of endocrine response in breast cancer by immunocytochemical detection of oestrogen receptors in fine needle aspirates. *Lancet*, **ii,** 701.

FLOWERS, J.L., COX, E.B., GEISINGER, K.R. & 4 others (1985). Use of monoclonal antioestrogen receptor antibody to evaluate oestrogen receptor content in fine needle aspiration breast biopsies. *Ann. Surg.*, **203,** 250.

FORREST, A.P.M., LEVACK, P.A., CHETTY, U. & 4 others (1986). A human tumour model. *Lancet*, **ii,** 840.

GREEN, G.L., FITCH, F.W. & JENSEN, E.V. (1980). Monoclonal antibodies to estrophilin: Probes for the study of oestrogen receptors. *Proc. Natl Acad. Sci. (USA)*, **77,** 157.

HAWKINS, R.A. (1985). Receptors in the management of breast cancer. *Br. J. Hosp. Med.*, **34,** 160.

HAWKINS, R.A., BLACK, R., STEELE, R.J.C., DIXON, J.M.J. & FORREST, A.P.M. (1981). Oestrogen receptor concentration in primary breast cancer and axillary node metastases. *Breast Cancer Res. Treatment*, **1,** 245.

HAWKINS, R.A., HILL, A., FREEDMAN, B., GORE, S.M., ROBERTS, M.M. & FORREST, A.P.M. (1977). Reproducibility of measurements of oestrogen receptor concentration in breast cancer. *Br. J. Cancer*, **36,** 355.

HAWKINS, R.A., SANGSTER, K. & KRAJEWSKI, A. (1986). Histochemical detection of oestrogen receptors in breast carcinoma: A successful technique. *Br. J. Cancer*, **53,** 407.

KING, W.J., DE SOMBRE, E.R., JENSEN, E.V. & GREENE, G.L. (1985). Comparison of immunocytochemical and steroid-binding assays for oestrogen receptor in human breast tumours. *Cancer Res.*, **45,** 293.

McCARTY, K.S., MILLER, L.S., COX, E.B., KONRATH, J & McCARTY, K.S. (1985). Estrogen receptor analyses: Correlation of biochemical and immunohistochemical methods using monoclonal antireceptor antibodies. *Arch. Pathol. Lab. Med.*, **109,** 716.

McLELLAND, R.A., BERGER, U., MILLER, L.S., POWLES, T.J. & COOMBES, R.C. (1986). Immunocytochemical assay for oestrogen receptor in patients with breast cancer: Relationship to biochemical assay and outcome of therapy. *J. Clin. Oncol.*, **4,** 1171.

McLELLAND, R.A., BERGER, U., WILSON, P. & 5 others (1987). Presurgical determination of oestrogen receptor status using immunocytochemically stained fine needle aspirate smears in patients with breast cancer. *Cancer Res.*, **47,** 6118.

McLELLAND, R.A. & COOMBES, R.C. (1985). Immunocytochemical assay for oestrogen receptors (ERICA): Compatability with a steroid-binding assay and value in predicting outcome of endocrine therapy in metastatic breast cancer. In *Proceedings International Association for Breast Cancer Research Biennial Conference* p. 79 (*Abstract 2–19*).

SENBANJO, R.O., MILLER, W.R. & HAWKINS, R.A. (1986). Variations in steroid receptors and cyclic AMP binding proteins across human breast cancers: Evidence for heterogeneity. *Br. J. Cancer*, **54:** 127.

WEINTRAUB, J., WEINTRAUB, D., REDARD, M. & VASSILAKOS, P. (1987). Evaluation of estrogen receptors by immunocytochemistry on fine needle aspiration biopsy specimens from breast tumours. *Cancer*, **60,** 1163.


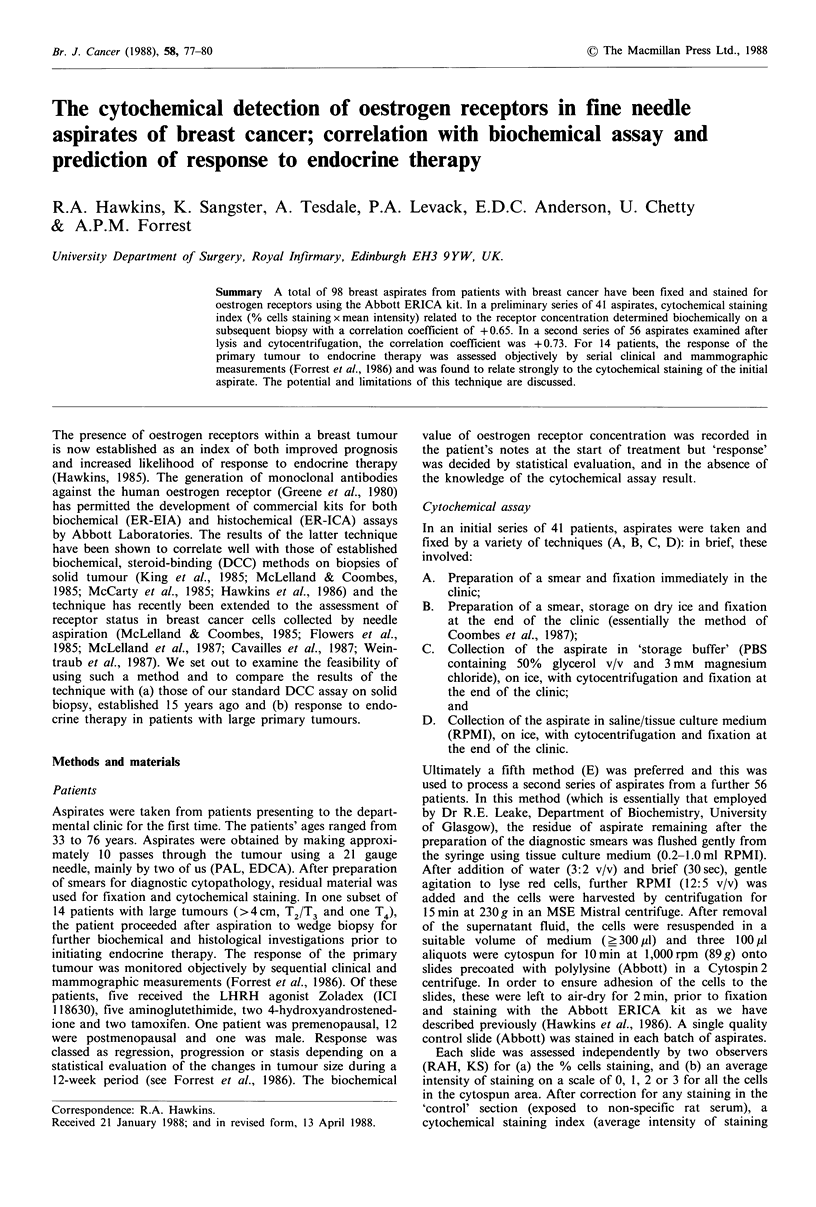

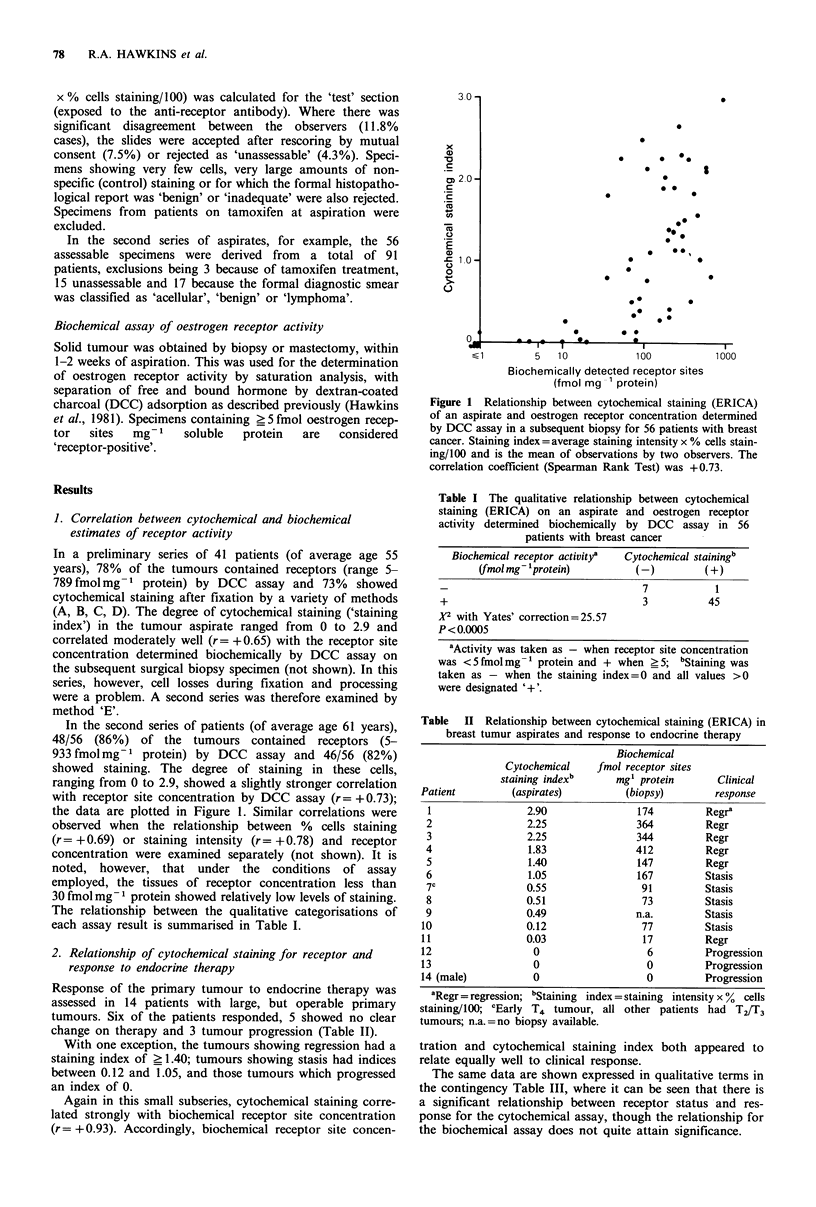

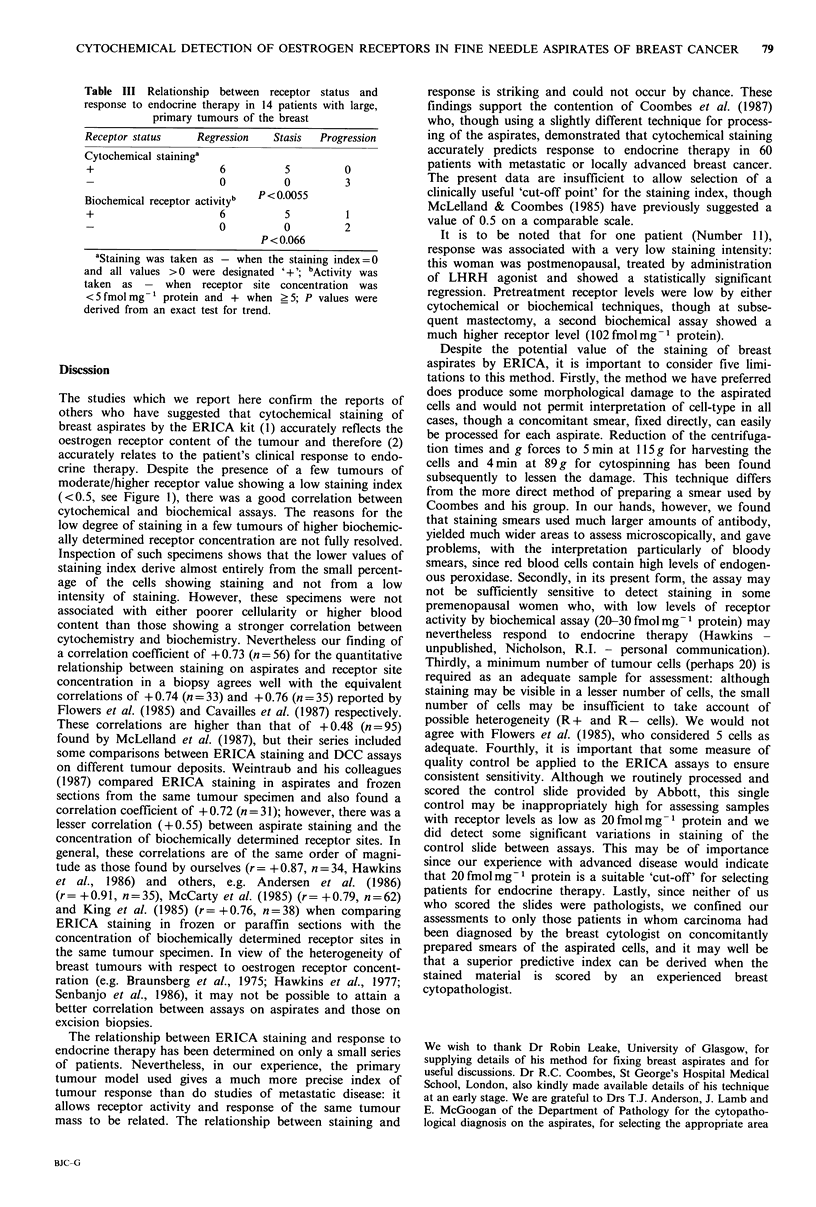

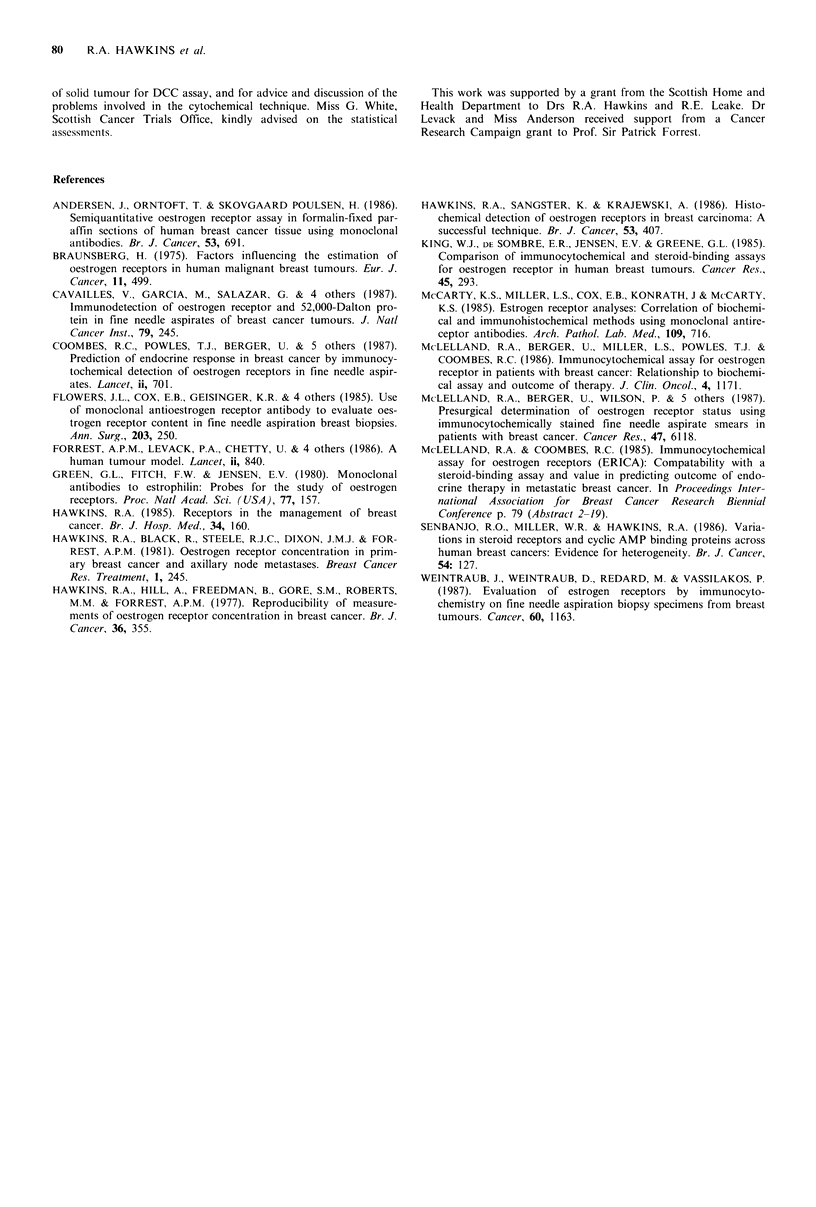


## References

[OCR_00530] Andersen J., Orntoft T., Poulsen H. S. (1986). Semiquantitative oestrogen receptor assay in formalin-fixed paraffin sections of human breast cancer tissue using monoclonal antibodies.. Br J Cancer.

[OCR_00536] Braunsberg H. (1975). Factors influencing the estimation of oestrogen receptors in human malignant breast tumours.. Eur J Cancer.

[OCR_00541] Cavailles V., Garcia M., Salazar G., Domergue J., Simony J., Pujol H., Rochefort H. (1987). Immunodetection of estrogen receptor and 52,000-dalton protein in fine needle aspirates of breast cancer tumors.. J Natl Cancer Inst.

[OCR_00547] Coombes R. C., Powles T. J., Berger U., Wilson P., McClelland R. A., Gazet J. C., Trott P. A., Ford H. T. (1987). Prediction of endocrine response in breast cancer by immunocytochemical detection of oestrogen receptor in fine-needle aspirates.. Lancet.

[OCR_00553] Flowers J. L., Burton G. V., Cox E. B., McCarty K. S., Dent G. A., Geisinger K. R., McCarty K. S. (1986). Use of monoclonal antiestrogen receptor antibody to evaluate estrogen receptor content in fine needle aspiration breast biopsies.. Ann Surg.

[OCR_00559] Forrest A. P., Levack P. A., Chetty U., Hawkins R. A., Miller W. R., Smyth J. F., Anderson T. J. (1986). A human tumour model.. Lancet.

[OCR_00563] Greene G. L., Fitch F. W., Jensen E. V. (1980). Monoclonal antibodies to estrophilin: probes for the study of estrogen receptors.. Proc Natl Acad Sci U S A.

[OCR_00578] Hawkins R. A., Hill A., Freedman B., Gore S. M., Roberts M. M., Forrest A. P. (1977). Reproducibility of measurements of oestrogen-receptor concentration in breast cancer.. Br J Cancer.

[OCR_00568] Hawkins R. A. (1985). Receptors in the management of breast cancer.. Br J Hosp Med.

[OCR_00584] Hawkins R. A., Sangster K., Krajewski A. (1986). Histochemical detection of oestrogen receptors in breast carcinoma: a successful technique.. Br J Cancer.

[OCR_00589] King W. J., DeSombre E. R., Jensen E. V., Greene G. L. (1985). Comparison of immunocytochemical and steroid-binding assays for estrogen receptor in human breast tumors.. Cancer Res.

[OCR_00595] McCarty K. S., Miller L. S., Cox E. B., Konrath J., McCarty K. S. (1985). Estrogen receptor analyses. Correlation of biochemical and immunohistochemical methods using monoclonal antireceptor antibodies.. Arch Pathol Lab Med.

[OCR_00601] McClelland R. A., Berger U., Miller L. S., Powles T. J., Coombes R. C. (1986). Immunocytochemical assay for estrogen receptor in patients with breast cancer: relationship to a biochemical assay and to outcome of therapy.. J Clin Oncol.

[OCR_00607] McClelland R. A., Berger U., Wilson P., Powles T. J., Trott P. A., Easton D., Gazet J. C., Coombes R. C. (1987). Presurgical determination of estrogen receptor status using immunocytochemically stained fine needle aspirate smears in patients with breast cancer.. Cancer Res.

[OCR_00621] Senbanjo R. O., Miller W. R., Hawkins R. A. (1986). Variations in steroid receptors and cyclic AMP binding proteins across human breast cancers: evidence for heterogeneity.. Br J Cancer.

[OCR_00627] Weintraub J., Weintraub D., Redard M., Vassilakos P. (1987). Evaluation of estrogen receptors by immunocytochemistry on fine-needle aspiration biopsy specimens from breast tumors.. Cancer.

