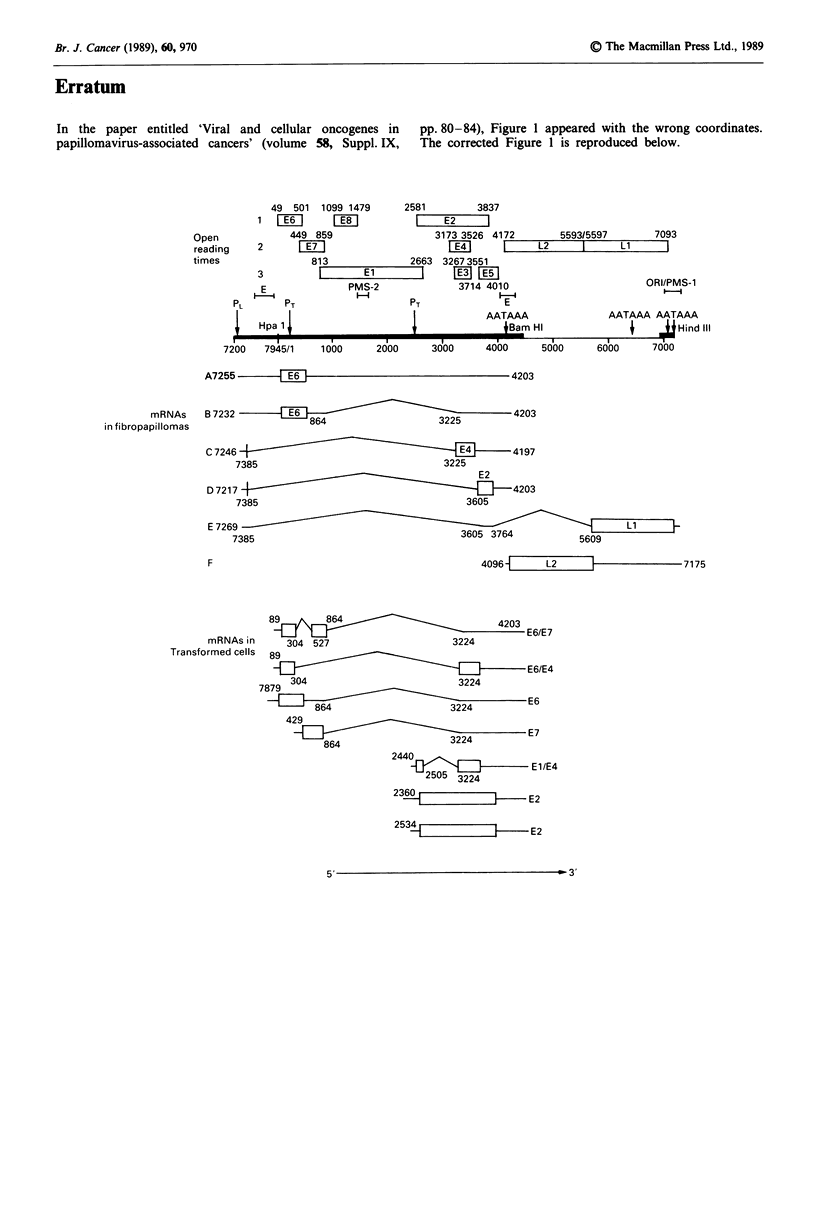# Erratum

**Published:** 1989-12

**Authors:** 


					
Br. J. Cancer (1989), 60, 970

?) The Macmillan Press Ltd., 1989

Erratum

In the paper entitled 'Viral and cellular oncogenes in  pp. 80-84), Figure 1 appeared with the wrong coordinates.
papillomavirus-associated cancers' (volume 58, Suppl. IX,  The corrected Figure 1 is reproduced below.

49   501  1099 1479        2581           3837

1 E6                                 E2

449  859                      3173 3526  4172          5593/5597          7093
2         E7                            E                L2               Li

813                 2663   3267 3551

3               ~~~~~El             E3  ES_

E                 PMS-2                 3714 4010                              ORI/PM

PT                        PT                 E

AATAAA                   AATAAA AATA

Hpa_ 1                     _Bam HI                                                Ai

2000    3000      4000      500I  I  I         7

2000      3000     4000       SOQO     6000      7000

7

7200   7945/1     1000

IS-1

AAA

Hind III

mRNAs
in fibropapillomas

A7255             E                                              4203

B 7232                                                           4203

864                 ~~~~3225        40

C 7246                                                           4197

7385                                        3225

E2

D7217                               }4203

7385                                             3605

E7269                                                 3605 3764

F

4096 i     L2      1                7175

89       864                       4203

E6/E7
mRNAs in    304 527                  3224
Transformed cells 89

{}                     -    }E      /E6/E4
7879304                        3224

864 8   X            3224        E6
429

3224    ~   E7
864                3224

2440

2360EI               E2

2534 L                   E2

9; I                                                                                                                 0.     3'

Open

reading
times

PL